# Transcriptomic Insights into Molecular Response of Butter Lettuce to Different Light Wavelengths

**DOI:** 10.3390/plants13121582

**Published:** 2024-06-07

**Authors:** Yongqi Liang, Xinying Weng, Hao Ling, Ghazala Mustafa, Bingxian Yang, Na Lu

**Affiliations:** 1 Shanxi Qingmei Biotechnology Company Limited, Baoji 721000, China; 2 College of Life Sciences and Medicine, Zhejiang Sci-Tech University, Hangzhou 310000, China; 18845132346@163.com (X.W.); 13955691927@163.com (H.L.); xianyb@126.com (B.Y.); 3 Department of Plant Sciences, Faculty of Biological Sciences, Quaid-i-Azam University, Islamabad 45320, Pakistan; mghazala@qau.edu.pk; 4 Center for Environment, Health and Field Sciences, Chiba University, 6-2-1 Kashiwanoha, Kashiwa 277-0882, Japan

**Keywords:** butter lettuce, physiology, nutritional content, transcriptomics, light spectrum

## Abstract

Lettuce is a widely consumed leafy vegetable; it became popular due to its enhanced nutritional content. Recently, lettuce is also regarded as one of the model plants for vegetable production in plant factories. Light and nutrients are essential environmental factors that affect lettuce growth and morphology. To evaluate the impact of light spectra on lettuce, butter lettuce was grown under the light wavelengths of 460, 525, and 660 nm, along with white light as the control. Plant morphology, physiology, nutritional content, and transcriptomic analyses were performed to study the light response mechanisms. The results showed that the leaf fresh weight and length/width were higher when grown at 460 nm and lower when grown at 525 nm compared to the control treatment. When exposed to 460 nm light, the sugar, crude fiber, mineral, and vitamin concentrations were favorably altered; however, these levels decreased when exposed to light with a wavelength of 525 nm. The transcriptomic analysis showed that co-factor and vitamin metabolism- and secondary metabolism-related genes were specifically induced by 460 nm light exposure. Furthermore, the pathway enrichment analysis found that flavonoid biosynthesis- and vitamin B6 metabolism-related genes were significantly upregulated in response to 460 nm light exposure. Additional experiments demonstrated that the vitamin B6 and B2 content was significantly higher in leaves exposed to 460 nm light than those grown under the other conditions. Our findings suggested that the addition of 460 nm light could improve lettuce’s biomass and nutritional value and help us to further understand how the light spectrum can be tuned as needed for lettuce production.

## 1. Introduction

Light is one of the most important environmental signals underlying plant growth patterns, and photons with wavelengths between 400 and 750 nm are essential for plant growth and development through controlling photosynthetic activity and regulating morphological adaptations and secondary metabolism [1]. Plants perceive different light signals due to the presence of various photoreceptors [2]. A large amount of research has been performed to explore the role of light-emitting diode (LED) lights on plant growth, morphology, production, and nutrient quality [3,4,5]. The UV spectrum plays a prominent role in inducing flavonoid biosynthesis in plants by activating flavonoid-related genes [6]. Longer wavelengths (red light and far-red light) can increase the content of soluble sugars in fruits [7]. Chlorophyll and carotenoid levels are decreased upon exposure to far-red light conditions [8]. On the other hand, higher levels of far-red light exposure improved the sugar content, which has important significance for human health [9,10]. Green light and yellow light may also play a role in altering plant physiology and growth [11]. Studies illustrated that white light increased the dry leaf weight and reduced the tuber weight of potato plantlets relative to the effect of blue or red light, while blue light increased the leaf area and anthocyanin content [12]. However, the molecular basis of the effect of red, blue, green, and white light on the formation of various quality traits in plants has been not well explained.

Lettuce (*Lactuca sativa* var. longifolia) is the most popular leafy vegetable grown indoors and it can be eaten raw or cooked at various stages of growth [13]. This plant is a source of vitamins and metabolites such as phenolic compounds related to human health [14]. Lettuce growth in greenhouses is regulated by additive light application, and is considered a model crop because of its sensitivity to light quality [15]. The lettuce plant is best adaptable to grow under a controlled environment [16]. Some lettuce varieties exhibit shade tolerance responses on exposure to supplemental far-red light, such as increased leaf expansion and seedling growth [17]. The 400–750 nm wavelength range has recently been referred to as extended photosynthetically active radiation [18]. In lettuce, photosynthetically active radiation flux did not show any interaction with the far-red fraction [19]. On the other hand, at high photosynthetically active radiation fluxes, the leaf area and dry mass increased under the far-red fraction; conversely, at lower photosynthetically active radiation fluxes, there was a drop in lettuce yield [20]. Exposure to far-red light enhanced overall plant growth with increased accumulation of bioactive compounds in the lettuce [21,22]. Far-red light exposure increased the light use efficiency through enhancing the leaf area; therefore, plants capture more light for photosynthesis [23]. It was shown that blue illumination increased the dark respiration rate and cyclic electron flow around photosystem I of lettuce, but decreased the rate of the photosynthetic linear electron flow, as well as various plant growth parameters in comparison to red illumination [24]. A recent study showed that an increased illumination duration can stimulate the dry weight accumulation of lettuce [1]. The next-generation sequencing was performed to analyze the differential expression of genes in lettuce leaves in response to different light spectra [25]. A transcriptomic analysis was also performed to identify the circadian clock-related genes in lettuce [26]. In red leaf lettuce, the expression of anthocyanin biosynthesis-related genes was detected by transcriptomic technology under different light intensities [27]. However, using the transcriptome data to determine the molecular basis of lettuce’s variable nutritional quality and growth under different light spectra was lacking. In this study, the response mechanisms of lettuce to different light wavelengths were investigated using morphological, mineralogical, nutritional, and transcriptomic strategies. The aim was to provide valuable insights into the optimal light wavelength and nutritional solution to improve the quality and yield of lettuce.

## 2. Results

### 2.1. Morphological Response of Butter Lettuce on Exposure to Various Light Wavelengths

**Figure 1:**
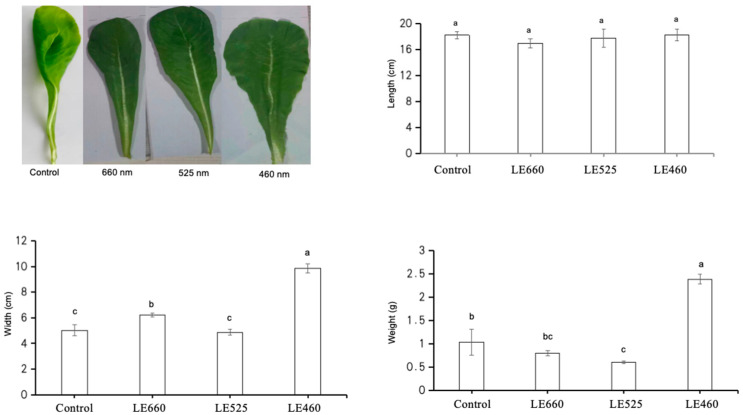
Morphological characteristics of leaves of butter lettuce grown under different light wavelengths. Data are presented as mean ± SD of three independent biological replicates. The same letters on bar graph indicate no significant differences among the samples based on one-way ANOVA (*p* < 0.05).

In order to evaluate the impact of different light wavelengths on the growth of butter lettuce, the leaf fresh weight, length, and width were measured. The results indicated that the application of different light wavelengths led to obvious morphological changes in the lettuce. All treatments stimulated lettuce plant growth: the leaf fresh weight, length, and width increased considerably on exposure to the different light wavelengths. The leaf fresh weight was significantly increased on exposure to 460 nm light while it decreased under 525 nm light compared to the control treatment (Figure 1). The leaf length and width also significantly increased under 460 nm exposure (17.8 + 1.1, 9.33 + 0.5) while they decreased under 525 nm exposure (16.9 + 0.3, 4.7 + 0.1) compared to the control treatment (Figure 1).

### 2.2. Light Wavelength Impacts the Crude Fiber and Soluble Sugar Levels in Butter Lettuce

**Figure 2:**
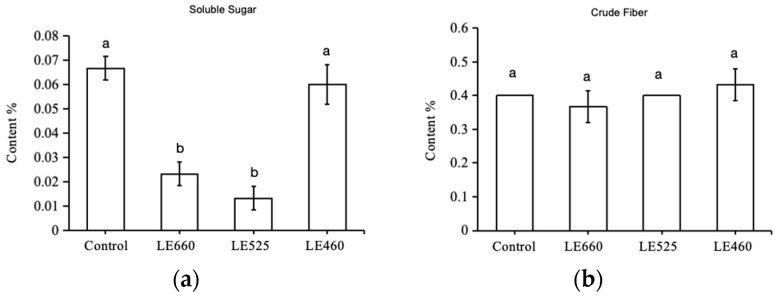
Estimation of soluble sugar (**a**) and crude fiber (**b**) content in butter lettuce leaves. Data are presented as mean ± SD of three independent biological replicates. The same letters on bar graph indicate no significant differences among the samples based on one-way ANOVA (*p* < 0.05).

To evaluate the impact of different light wavelengths on the biochemical aspects of butter lettuce, the soluble sugars and crude fiber from treated and control leaves were examined. The soluble sugar content of lettuce in response to various light wavelengths is shown in Figure 2a. The results indicated that different light wavelengths had a considerable impact on the soluble sugar content. The soluble sugar content was severely reduced when exposed to 525 nm light while it increased when exposed to 460 nm light (Figure 2a). The crude fiber content of the lettuce was also evaluated after exposure to different light wavelengths. The content did not change significantly upon exposure to 525 nm light, while there was a slight increase upon exposure to 460 nm light (Figure 2b).

### 2.3. Mineral Analysis of Butter Lettuce upon Exposure to Different Light Wavelengths

**Figure 3:**
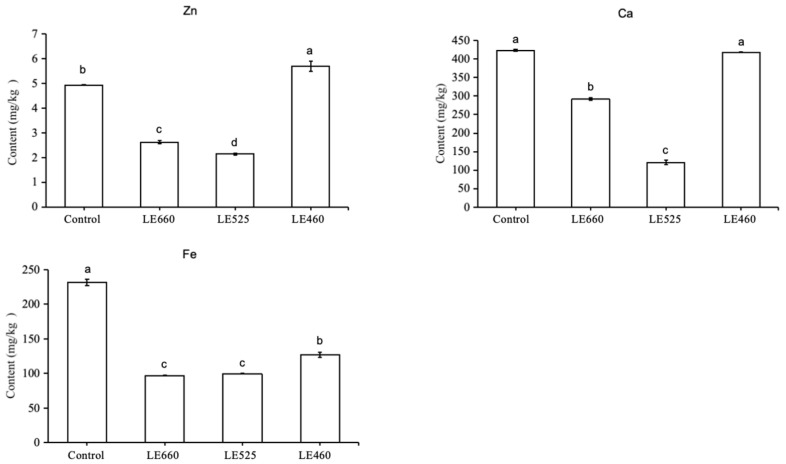
The metal ion content in leaves of butter lettuce. Data are presented as mean ± SD of three independent biological replicates. The same letters on bar graph indicate no significant differences among the samples based on one-way ANOVA (*p* < 0.05).

To evaluate the impact of different light wavelengths on the biochemical aspects of butter lettuce, the mineral contents from treated and control leaves were examined. The different light wavelengths exhibited a significant effect on the total Zn, Ca, and Fe content in the lettuce (Figure 3). The total Zn content was highest at a wavelength of 460 nm and lowest at 525 nm. The total Ca content in the lettuce increased under 460 nm light while it was reduced under 525 nm light exposure compared to the control treatment. The Fe content was negatively impacted by all light wavelengths compared to the control treatment. However, the Fe content was higher when grown under 460 nm light while it was lower when exposed to 525 nm and 660 nm light (Figure 3).

### 2.4. Light-Mediated Variations in Differentially Expressed Genes of Butter Lettuce Leaves

**Figure 4:**
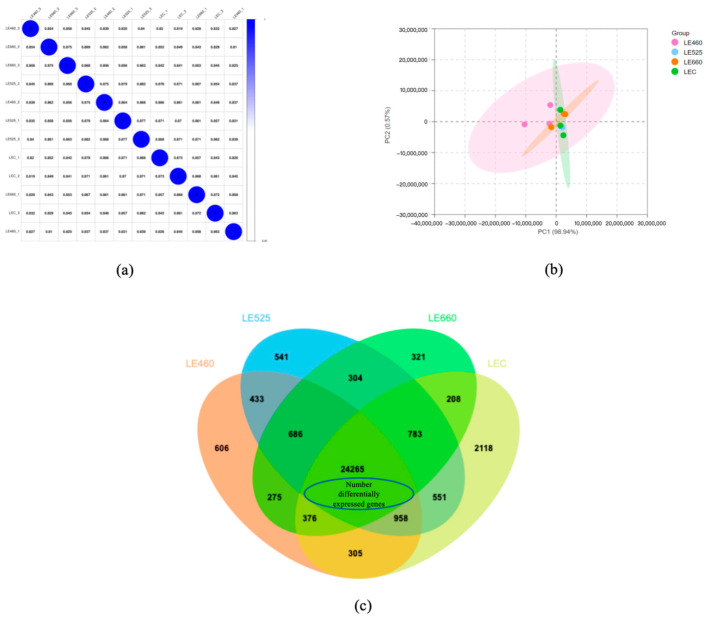
Transcriptomic analysis of leaves under different light wavelengths. (**a**) The correlation analysis between samples; (**b**) the PCA analysis of samples; (**c**) Venn diagram showing the overlap of identified genes in samples. The number of differentially expressed genes that were commonly identified is shown in the center of the Venn diagram.

In the present study, to reveal the molecular mechanism underlying the lettuce response to different light wavelengths, a transcriptomic analysis was carried out. The number of identified genes under the control, 460 nm, 525 nm, and 660 nm light conditions were 29,564, 27,904, 30,788, and 28,821, respectively. We further performed a pairwise correlation among all treatments and found an average pairwise correlation coefficient of 0.8608 (Figure 4a). The principal component plot of the expressed genes from all three treatments showed two major factors: PC1 (98.94%) and PC2 (0.57%) (Figure 4b). A Venn diagram (Figure 4c) was constructed to summarize the number of identified genes in response to the light wavelengths of 460, 525, and 660 nm. Some genes were commonly regulated among the different light wavelengths; however, others were specific to a particular light wavelength. The Venn diagram analysis revealed that 24,265 genes were commonly expressed among the three treatments in lettuce leaves, whereas 606 genes, 541, 321, and 2118 genes were specifically under 460 nm, 525 nm 660 nm light wavelengths and in the control, respectively. Furthermore, 4949 genes were identified to be significantly changed under all the applied light wavelengths.

### 2.5. Cluster and Gene Ontology Analyses of Differentially Expressed Genes in Lettuce Leaves upon Exposure to Different Light Wavelengths 

**Figure 5:**
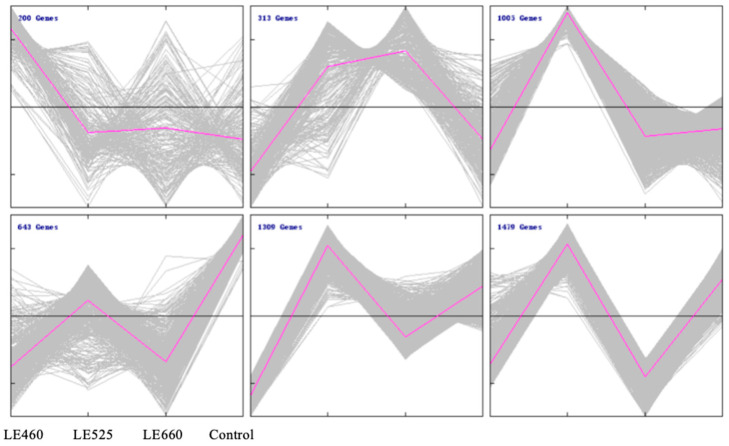
Clusters of differentially expressed genes. Differentially expressed genes were clustered and are represented as line charts with the help of Mev. Six clusters were identified. The x-axis represents the samples from plants grown under different light wavelengths. The y-axis indicates the normalized gene expression level. In each cluster, the black line indicates the zero line while the purple line indicates the average expression level. The number at the upper left corner represents the number of DEGs in each cluster.

To further analyze the abundance profiles of the differentially expressed 4949 genes in lettuce leaves exposed to different light wavelengths compared to the control treatment, hierarchical clustering was performed. Six clusters were produced and 200, 313, 1005, 643, 1309, and 1479 genes were contained in clusters 1–6, respectively (Figure 5). The genes in cluster 1 were significantly induced by 460 nm light exposure compared with the other light wavelengths. The genes in cluster 2 were upregulated under both 525 and 660 nm light. However, cluster 3 and cluster 5 contained genes that were specifically induced by 525 nm light exposure.

**Figure 6:**
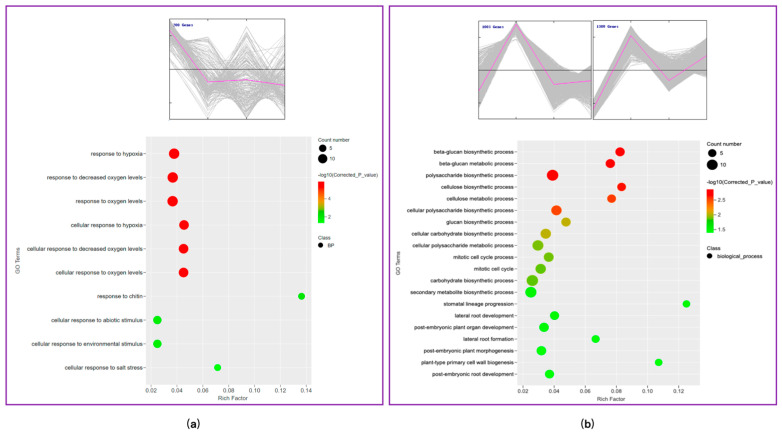
The GO enrichment analysis of genes. (**a**) GO enrichment analysis exposed to 460 nm light; (**b**) GO enrichment analysis exposed to 525 nm light.

In order to understand the functional role of these identified genes, a gene ontology analysis of the 200 genes in cluster 1 was performed (Figure 6a). The functional categorization indicated that the genes involved in the response to hypoxia (10 genes) and the response to decreased oxygen levels (10 genes) were up-regulated. This up-regulation of genes is associated with a stress response upon exposure to 460 nm light. The functional categorization of the 1003 and 1309 genes in cluster 3 indicated that the genes responsible for beta-glucan biosynthetic processing (10 genes) and polysaccharide biosynthetic processing (10 genes) were up-regulated in response to 525 nm light.

### 2.6. Gene Ontology Analysis of Common DEGs in Lettuce Leaves Exposed to Different Light Wavelengths 

**Figure 7:**
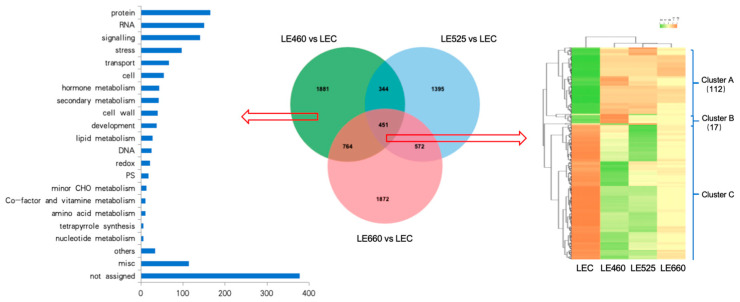
Functional categorization and enrichment of genes. The comparisons between LE460 nm/LE525 nm/LE660 nm and LEC were performed to identify the differentially expressed genes. Gene functions were predicted and categorized using MapMan bin codes. The cluster analysis of the common differentially expressed genes produced three clusters.

To perform a more in-depth analysis, the genes were analyzed by comparing the wavelengths of 460 nm, 525 nm, and 660 nm to LEC through a Venn diagram (Figure 7). In the Venn diagram, 1881 genes showed specific differential expression upon 460 nm light exposure while 1395 genes were induced by 525 nm light and 1872 genes were specifically induced by 660 nm light exposure. The Venn diagram also indicated that the 451 genes were commonly induced in lettuce leaves on exposure to all light wavelengths. The gene ontology analysis of the 1881 genes induced by 460 nm light indicated that the genes involved in signaling, stress, and hormone metabolism were significantly induced. A hierarchical clustering analysis of the 451 commonly identified genes also highlighted the up- and down-regulation of various genes. The green and red colors indicate the up-regulated and down-regulated genes, respectively (Figure 7). A cluster analysis indicated that the genes in Cluster A (112 genes) and Cluster B (17 genes) were significantly up-regulated upon 460 nm exposure compared to the control treatment.

### 2.7. Pathway Analysis of Common Differentially Expressed Genes in Lettuce Leaves Exposed to Different Light Wavelengths 

**Figure 8:**
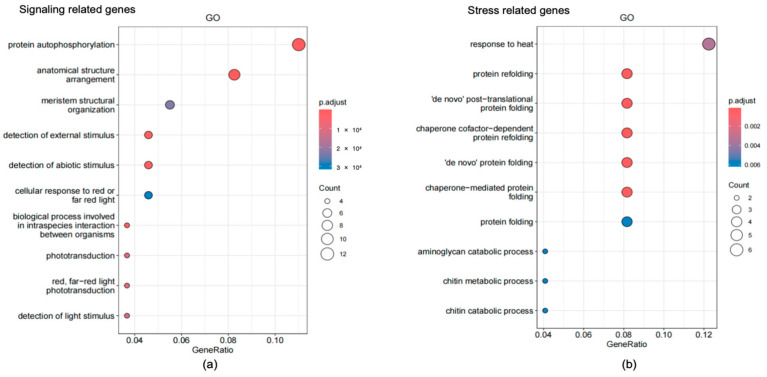
The KEGG enrichment analysis of genes. (**a**) KEGG enrichment analysis result of signaling-related genes; (**b**) KEGG enrichment analysis result of stress related genes.

The scatter plot pathway analysis of signaling-related genes indicated that most of the genes were enriched in protein autophosphorylation (12 genes) and anatomical structure arrangements (10 genes) (Figure 8a). Phototransduction, red/far-red light phototransduction, and detection of light stimulus-related genes were also activated by 460 nm light exposure. Stress-related genes were mostly involved in the response to heat, protein refolding, protein folding, and the aminoglycan catabolic process (Figure 8b).

**Figure 9:**
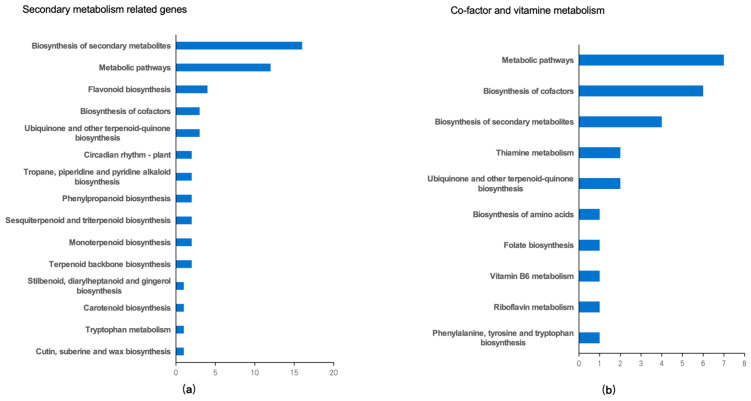
Function analysis of genes. Gene functions were predicted and categorized using MapMan bin codes. (**a**) Function analysis result of secondary metabolism related genes; (**b**) Function analysis result of Co-factor and vitamine metabolism genes.

The pathway analysis of genes involved in secondary metabolism showed that the genes related to flavonoid biosynthesis were up-regulated (Figure 9a). The pathway analysis of co-factor and vitamin metabolism-related genes indicated that the genes involved in vitamin B6 metabolism were up-regulated upon 460 nm light exposure (Figure 9b).

### 2.8. Vitamin Content of Butter Lettuce upon Exposure to Different Light Wavelengths

**Figure 10:**
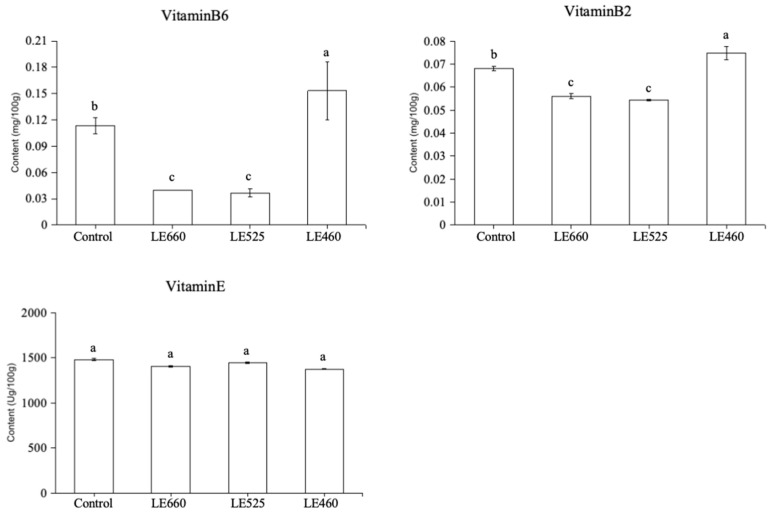
The vitamin content in leaves grow under different light wavelengths. Data are presented as mean ± SD of three independent biological replicates. The same letters on bar graph indicate no significant differences among the samples based on one-way ANOVA (*p* < 0.05).

The vitamin content in lettuce was significantly affected upon exposure to the various light wavelengths (Figure 9b); a confirmatory experiment was performed to check the variation in vitamin content when grown at different light wavelengths. The vitamin B6 content was significantly reduced when exposed to 660 and 525 nm light compared to the control treatment (Figure 10). On the contrary, there was a significant increase in vitamin B6 content upon exposure to 460 nm light compared to the control treatment. The vitamin B2 content was also reduced upon 660 nm and 525 nm light exposure while it increased upon 460 nm exposure compared to the control treatment. However, the vitamin E content remained unchanged when grown under all the light wavelengths.

### 2.9. Chlorophyll and Anthocyanin Content of Lettuce Exposed to Different Light Wavelengths

**Figure 11:**
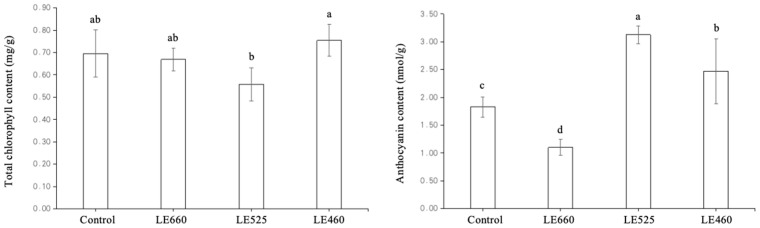
The chlorophyll and anthocyanin content in lettuce grown under different light wavelengths. Data are presented as mean ± SD of three independent biological replicates. The same letters on bar graph indicate no significant differences among the samples based on one-way ANOVA (*p* < 0.05).

Confirmatory experiments were performed to determine the influence of the different light wavelengths on the chlorophyll and anthocyanin content of lettuce. The total chlorophyll content was slightly increased and decreased upon 460 nm and 525 nm light exposure compared to the control treatment, respectively (Figure 11). It was interesting that the anthocyanin content slightly increased upon 460 nm and 525 nm light exposure by 1.35 and 1.71 times compared to the control treatment. However, the anthocyanin content significantly decreased in response to 660 nm light.

## 3. Discussion

### 3.1. Regulation of Hypoxia in Lettuce upon Exposure to Different Light Wavelengths

Lighting patterns that include 12 h of blue-light mono-irradiation promote leaf elongation, probably owing to the phytochrome reaction in lettuce [28]. In another study, green light enhanced the growth of lettuce in comparison to red and blue light [29]. In lettuce, the highest levels of shoot fresh/dry weight were reported under red/blue light compared to other treatments [30], while alternating red/blue light caused an increase in lettuce yield [31]. Various combinations of light also positively or negatively impact the growth of lettuce. The combination of red and blue light enhanced the biomass accumulation in lettuce [32,33]. In lettuce, exposure to continuous light of different wavelengths leads to the accumulation of reactive oxygen species and lipid peroxidation [34]. Plants have developed different strategies to combat the damage caused by different light wavelengths. Plants tend to move their leaves in order to avoid light-induced damage. It is worth mentioning here that the leaf weight and width of lettuce was reduced when grown under 660 nm light compared to the other light wavelengths, indicating the plants’ implementation of the avoidance strategy (Figure 2). There is an increase in the generation of reactive oxygen species under stress conditions. Plants try to reduce reactive oxygen species levels through the regulation of different scavenging enzymes and antioxidants. Plants need to accurately regulate gene expression to appropriately coordinate their development in response to changing environmental conditions. Such regulation occurs at the direct transcriptional level through the action of transcription factors. In lettuce, the blue light (460 nm) exposure up-regulated the transcripts related to stress responses while red light (630 nm) exposure up-regulated oxidation–reduction-related transcripts [25].

Hypoxia in plants occurs frequently as a result of O_2_ diffusion limitations and the rapid consumption rates in tissues with high energy demands [35,36,37]. Hypoxia pre-adaptation and the induction of stomatal closure play considerable roles in the regulation of phosphine phytotoxicity in Arabidopsis thaliana [38]. Hypoxia promoted adventitious root elongation mediated by ERFVII transcription factors in Arabidopsis thaliana [39]. Hypoxia activated the endoplasmic reticulum-anchored Arabidopsis ANAC-related module to enable fast transcriptional reprogramming in Arabidopsis in response to low-oxygen stress [40]. In the present study, more transcripts related to hypoxia were up-regulated. This indicates that lettuce might promote hypoxia signaling after monitoring the oxygen availability and adjust its growth and metabolism accordingly when under light stress.

### 3.2. Vitamin Regulation in Lettuce Exposed to Different Light Wavelengths 

In lettuce, exposure to diffuse light can damage the vitamin C content [41]. In lettuce, the exposure to a light/dark cycle induced the production of vitamin C [42]. In another study, red light enhanced the vitamin C content compared to the other treatments in the study [43]. In the present study, blue light enhanced the accumulation of vitamin C in lettuce, which is in agreement with a previous study [44].

Vitamin B6 is an important cofactor in many biochemical processes, most importantly in amino acid metabolism [45]. In Arabidopsis, a deficiency in vitamin B6 was linked with reduced tolerance to photo-oxidative stress, leading to the enhanced oxygen levels and lipid peroxidation after high light exposure [46]. Yeast and animal cell cultures, on exposure to exogenous vitamin B6 molecules, were used to demonstrate the antioxidant properties of vitamin B6 [47,48,49,50]. Similar to vitamin B6, exogenously administered vitamin B6 has been shown to shield plant protoplasts from O_2_-induced cell death [51]. These findings raise the question of whether plants use vitamin B6 in the antioxidant defense against reactive oxygen species. In the present study, the cofactor and vitamin-related transcripts were upregulated upon exposure to 460 nm light. These results were confirmed by the estimation of the vitamin B2, B6, and E content in the lettuce leaves grown under different light wavelengths. The vitamin B6 content was increased under 460 nm light compared to the other wavelengths. These results suggest that the levels of vitamin B6 were increased to scavenge the reactive oxygen species to reduce the effects of light stress.

### 3.3. Regulation of Secondary Metabolism in Lettuce upon Exposure to Different Light Wavelengths

The ability of plants to produce metabolites is viewed as a strategy to cope with the stressful conditions produced by the exposure to changing growth environments, which results in alterations in the structure and function of different signaling pathways [52,53]. When cell cultures are exposed to biotic and abiotic factors and signaling molecules, the creation of a large number of secondary molecules—which are biosynthesized from primary metabolites and accumulate in plant cells—can be stimulated in an in vitro setting. Light plays a crucial function in encouraging plant development and activating or controlling plant metabolism. By releasing and accumulating different secondary metabolites, such as phenolic compounds, triterpenoids, and flavonoids, under light radiation, plants can respond to alterations in their environmental conditions. These compounds are reported to have economic value because of their antioxidant properties. The biosynthetic processes of a developing plant need light to function. The photoperiod, strength, and quality are the main determinants of effects of light radiation [54,55]. In lettuce, the secondary metabolite-related transcripts were upregulated upon exposure to the different light wavelengths [25]. In Rhododendron molle, de novo transcriptome assembly was used to identify genes involved in the regulation of secondary metabolite synthesis [56]. To investigate the impact of various light sources on the regulation of the genes involved in leaf aging in leaf lettuce, a gene expression investigation utilizing RNA-Seq technology was recently carried out [57]. In the present study, the secondary metabolite-related transcripts were upregulated, indicating the regulation of flavonoid biosynthesis pathways by different light wavelengths.

## 4. Materials and Methods

### 4.1. Plant Treatment

Growing lettuce in a growth room uses different light spectra. These wavelengths include 460 nm, 525 nm, and 660 nm, along with mixed wavelengths which were used as the control treatment. The growth conditions were controlled at 28–30 °C and 70–80% relative humidity, with an 8 h (light)–16 h (dark) irradiation cycle.

Firstly, we placed the seeds in a 50-hole seedling tray, and then used a 1/2 ms substrate to propagate them periodically. After 5 days, the seeds began to germinate. We exposed the germinated lettuce to light with an initial intensity of 50 µmol m^−2^ s^−1^. Such lighting conditions help promote the early stages of seedling development.

Then, we gradually increased the light intensity to meet the energy needs necessary for lettuce growth. On day 14, the light intensity was increased to 100 µmol m^−2^ s^−1^. On day 21, the light intensity was increased to 400 µmol m^−2^ s^−1^. Such strong light irradiation can stimulate plants to participate in stronger photosynthesis and can promote the growth rate of lettuce. On day 45, we found that using different wavelengths had a great impact on plant growth. The leaves of the control and light-treated butter lettuce were collected as samples for further analyses.

### 4.2. RNA Sequencing

Total RNA was extracted from each collected plant sample using the RNA mini kit (Qiagen, Hilden, Germany). Gel electrophoresis and a Qubit analyzer (Thermo, Waltham, MA, USA) were used to evaluate the quality and integrity of the extracted RNA. For the RNA sequencing, 1 μg of extracted RNA was used for reverse transcription into double-stranded cDNA using the Smart-RT Enzyme (Takara, Japan) and then purified with magnetic beads to repair the ends of short fragments by adding a poly (A) tail and the sequencing connector. The cDNA from each group of three individuals (one per biological replicate) was pooled to build a sequencing library, which was purified and qualitatively and quantitatively assayed using gel electrophoresis and real-time PCR, respectively. Sequencing libraries were generated using the VAHTSTM Total RNA-seq (H/M/R) Library Prep Kit from Illumina^®^ (Vazyme Biotech Co., Ltd., Nanjing, China). The constructed libraries were sequenced as 151 bp paired-end reads using Illumina Novaseq6000 according to the manufacturer’s instructions by Genergy Biotechnology Co., Ltd. (Shanghai, China).

### 4.3. Reads Mapping and Transcript Assembly

From the raw pair-end reads, adapters and poor-quality bases were cut off with the help of Skewer (version 0.2.2). The clean reads were aligned to the reference genome downloaded from the Ensembl website (http://ensemblgenomes.org/, accessed on 5 November 2023) using STAR version 2.5.3a and a comprehensive gene annotation GTF file from Ensembl website. The mapped reads of each sample were assembled into transcripts based on the reference-guided assembly strategy using StringTie version 1.3.1 [58]. The assembled transcripts were merged with StringTie to obtain a non-redundant unified set of transcripts. The set of assembled transcripts was compared to Ensembl gene annotations using gffcompare version 0.9.9c [59].

### 4.4. Differential Expression Analysis

The expression levels of all annotated genes were quantified using StringTie (version 1.3.1). Differential expression analysis was performed using the DESeq2 R package (1.28.1) with the likelihood ratio test option [60]. Differentially expressed genes exhibiting greater than two-fold changes with adjusted *p*-values <= 0.05 were selected. If the gene DESeq2 normalized read count value was close to 0, log2 transformation was performed after adding 1. The expression values were visualized using the R package heatmap.

### 4.5. Function Annotation and Enrichment

The functional gene prediction was performed by converting the annotations to the Arabidopsis genome, considering the orthologous genes. Gene functions were categorized using Mercator 4 (https://plabipd.de/portal/mercator4, accessed on 17 November 2023) [61]. MapMan software version 3.7.0 (http://gabi.rzpd.de/projects/MapMan/, accessed on 21 November 2023) and the Kyoto Encyclopedia of Genes and Genomes (KEGG) database (http://www.genome.jp/kegg/, accessed on 4 December 2023) [62] were used for the pathway mapping of the identified genes [63]. Fold change ratios of the differential expressed genes were estimated with the help of a hierarchical clustering analysis. The cluster analysis was performed using the K-Means method in MeV (Multiple Experiment Viewer) (https://sourceforge.net/projects/mev-tm4/files/mev-tm4/ (accessed on 4 December 2023).

### 4.6. Determination of Metal and Non-Metal Elements

In this study, the common metal elements and non-metal elements were determined using the method of the National Standard of the People’s Republic of China (GB 5009.268-2016) [64]. An Agilent 7500c Inductively Coupled Plasma Mass Spectrometer (Agilent, Tokyo, Japan) was used in this experiment. The working principle of ICP-MS is as follows: Firstly, a high-frequency alternating current power source is used to convert gas into plasma. Then, under the effect of high temperature and the high-energy electric field generated by the plasma, the chemical bonds of atoms or ions in the sample are broken, and they are converted into charged ions. Subsequently, these ions are guided into the mass spectrometer through an acceleration channel.

In the mass spectrometer, the ions pass through a quadrupole system where they are separated based on their mass-to-charge ratio using different magnetic fields. Then, these ions enter an ion multiplier one by one. In the ion multiplier, the ions are further accelerated and collide with organic molecules, causing them to fragment into thermally stable atomic ions. Finally, these atomic ions are counted and their relative abundance is measured by a detector.

### 4.7. Pigment Content Determination

Chlorophyll determination was carried out according to the method described in [65]. Chlorophylls were extracted from 50 mg of leaves in 5 mL of 95% ethanol at 25 °C for 1 day (until the leaves turned white). The extraction solution was measured at 470 nm, 649 nm, and 665 nm using a UV-1800PC spectrophotometer (MAPADA, Shanghai, China). The chlorophyll content was calculated using the formulas Chlorophyll a (Ca) = 13.95 × D665 − 6.88 × D649; Chlorophyll b (Cb) = 24.96 × D649 − 7.32 × D665; Total chlorophyll = Ca + Cb.

Anthocyanins were extracted and determined using the method of Wang et al. [66] with little modification. Briefly, 50 mg of frozen leaves was grounded into powder in liquid nitrogen, sonicated with 3 mL of 0.1% methanol hydrochloride for 1 h, and then shaken overnight. After centrifugation at 2500× *g* for 10 min, 1 mL of the supernatant was mixed with 1 mL of water and the mixture was further mixed with 1 mL of chloroform to remove the chlorophyll. The resulting solution was measured at 530 nm for anthocyanin determination.

### 4.8. Statistical Analysis

All the statistical calculations were performed using SPSS statistical software (version 22.0; IBM, Armonk, NY, USA). One-way ANOVA followed by Tukey’s test was used to calculate the statistical significance between multiple groups. A *p*-value < 0.05 was considered statistically significant. Three independent biological replicates were used for each sample group.

## 5. Conclusions

Lettuce is the most popular leafy vegetable that is grown indoors and is a source of vitamins and metabolites such as phenolic compounds related to human health. In this study, a transcriptomic analysis was performed to evaluate the impact of the light spectrum on lettuce; butter lettuce was grown under the light wavelengths of 460, 525, and 660 nm, along with white light as the control treatment. The results showed that the leaf fresh weight and length/width were higher when grown at 460 nm while these measures were lower when grown at 525 nm compared to the control plants. When exposed to 460 nm light, the sugar, crude fiber, mineral, and vitamin levels were favorably altered; however, these levels decreased when exposed to 525 nm light. The functional enrichment of differentially expressed genes indicated that co-factor and vitamin metabolism- and secondary metabolism-related genes were specifically induced by 460 nm light exposure. The pathway enrichment analysis found that flavonoid biosynthesis- and vitamin B6 metabolism-related genes were significantly upregulated in response to 460 nm light exposure. The additional experiments demonstrated the vitamin B6 and B2 content was significantly higher in leaves grown under 460 nm light than those grown under the other conditions. These results indicate that the addition of 460 nm light could improve lettuce’s biomass and nutritional value.

## Data Availability

The original contributions presented in the study are included in the article, further inquiries can be directed to the corresponding authors.

## References

[1] Yudina L., Sukhova E., Gromova E., Mudrilov M., Zolin Y., Popova A., Nerush V., Pecherina A., Grishin A.A., Dorokhov A.A. (2023). Effect of duration of LED lighting on growth, photosynthesis and respiration in lettuce. Plants.

[2] Sims D.A., Gamon J.A. (2002). Relationships between leaf pigment content and spectral reflectance across a wide range of species, leaf structures and developmental stages. Remote Sens. Environ..

[3] Mohamed S.J., Rihan H.Z., Aljafer N., Fuller M.P. (2021). The Impact of Light Spectrum and Intensity on the Growth, Physiology, and Antioxidant Activity of Lettuce (*Lactuca sativa* L.). Plants.

[4] Kornpointner C., Martinez A.S., Marinovic S., Haselmair-Gosch C., Jamnik P., Schröder K., Löfke C., Halbwirth H. (2021). Chemical composition and antioxidant potential of *Cannabis sativa* L. roots. Ind. Crops Prod..

[5] SharathKumar M., Heuvelink E., Marcelis L.F.M. (2020). Vertical Farming: Moving from Genetic to Environmental Modification. Trends Plant Sci..

[6] Siipola S.M., Kotilainen T., Sipari N., Morales L.O., Lindfors A.V., Robson T.M., Aphalo P.J. (2015). Epidermal UV-A absorbance and whole-leaf flavonoid composition in pea respond more to solar blue light than to solar UV radiation. Plant Cell Environ..

[7] Ji Y., Nuñez Ocaña D., Choe D., Larsen D.H., Marcelis L.F.M., Heuvelink E. (2020). Far-red radiation stimulates dry mass partitioning to fruits by increasing fruit sink strength in tomato. New Phytol..

[8] Kong Y., Nemali K. (2021). Blue and Far-Red Light Affect Area and Number of Individual Leaves to Influence Vegetative Growth and Pigment Synthesis in Lettuce. Front. Plant Sci..

[9] Zou J., Fanourakis D., Tsaniklidis G., Woltering E.J., Cheng R., Li T. (2023). Far-red radiation during indoor cultivation reduces lettuce nutraceutical quality and shortens the shelf-life when stored at supra optimal temperatures. Postharvest Biol. Technol..

[10] Hmelak Gorenjak A., Cencič A. (2013). Nitrate in vegetables and their impact on human health. A review. Acta Aliment..

[11] Miao L., Zhang Y., Yang X., Xiao J., Zhang H., Zhang Z., Wang Y., Jiang G. (2016). Colored light-quality selective plastic films affect anthocyanin content, enzyme activities, and the expression of flavonoid genes in strawberry (*Fragaria* × *ananassa*) fruit. Food Chem..

[12] Xu J., Yan Z., Xu Z., Wang Y., Xie Z. (2018). Transcriptome analysis and physiological responses of the potato plantlets in vitro under red, blue, and white light conditions. 3 Biotech.

[13] Noumedem J.A.K., Djeussi D.E., Hritcu L., Mihasan M., Kuete V. (2017). Lactuca sativa. Medicinal Spices and Vegetables from Africa.

[14] Kim H.J., Fonseca J.M., Choi J.H., Kubota C. (2007). Effect of methyl jasmonate on phenolic compounds and carotenoids of romaine lettuce (*Lactuca sativa* L.). J. Agric. Food Chem..

[15] Lin K.H., Huang M.Y., Huang W.D., Hsu M.H., Yang Z.W., Yang C.M. (2013). The effects of red, blue, and white light-emitting diodes on the growth, development, and edible quality of hydroponically grown lettuce (*Lactuca sativa* L. var. *capitata*). Sci. Hortic..

[16] Shimizu H., Saito Y., Nakashima H., Miyasaka J., Ohdoi K. (2011). Light environment optimization for lettuce growth in plant factory. IFAC Proc. Vol..

[17] Park Y., Runkle E.S. (2017). Far-red radiation promotes growth of seedlings by increasing leaf expansion and whole-plant net assimilation. Environ. Exp. Bot..

[18] Zhen S., Bugbee B. (2020). Far-red photons have equivalent efficiency to traditional photosynthetic photons: Implications for redefining photosynthetically active radiation. Plant Cell Environ..

[19] Legendre R., van Iersel M.W. (2021). Supplemental Far-Red Light Stimulates Lettuce Growth: Disentangling Morphological and Physiological Effects. Plants.

[20] Kusuma P., Bugbee B. (2023). On the contrasting morphological response to far-red at high and low photon fluxes. Front. Plant Sci..

[21] Lee M.J., Son K.H., Oh M.M. (2016). Increase in biomass and bioactive compounds in lettuce under various ratios of red to far-red LED light supplemented with blue LED light. Hortic. Environ. Biotechnol..

[22] Meng Q., Kelly N., Runkle E.S. (2019). Substituting green or far-red radiation for blue radiation induces shade avoidance and promotes growth in lettuce and kale. Environ. Exp. Bot..

[23] Zou J., Zhang Y., Zhang Y., Bian Z., Fanourakis D., Yang Q., Li T. (2019). Morphological and physiological properties of indoor cultivated lettuce in response to additional far-red light. Sci. Hortic..

[24] Yudina L., Sukhova E., Mudrilov M., Nerush V., Pecherina A., Smirnov A.A., Dorokhov A.S., Chilingaryan N.O., Vodeneev V., Sukhov V. (2022). Ratio of intensities of blue and red light at cultivation influences photosynthetic light reactions, respiration, growth, and reflectance indices in lettuce. Biology.

[25] Kumar V., Sugumaran K., Al-Roumi A., Shajan A. (2022). De-novo transcriptome assembly and analysis of lettuce plants grown under red, blue or white light. Sci. Rep..

[26] Higashi T., Aoki K., Nagano A.J., Honjo M.N., Fukuda H. (2016). Circadian oscillation of the lettuce transcriptome under constant light and light-dark conditions. Front. Plant Sci..

[27] Zhang Y., Xu S., Cheng Y., Peng Z., Han J. (2018). Transcriptome profiling of anthocyanin-related genes reveals effects of light intensity on anthocyanin biosynthesis in red leaf lettuce. PeerJ.

[28] Jishi T., Matsuda R., Fujiwara K. (2021). Blue light monochromatic irradiation for 12 hours in lighting pattern with combinations of blue and red light elongates young cos lettuce leaves and promotes growth under high daily light integral. HortScience.

[29] Kim H.H., Goins G.D., Wheeler R.M., Sager J.C. (2004). Green-light supplementation for enhanced lettuce growth under red-and blue-light-emitting diodes. HortScience.

[30] Chen X.L., Wang L.C., Li T., Yang Q.C., Guo W.Z. (2019). Sugar accumulation and growth of lettuce exposed to different lighting modes of red and blue LED light. Sci. Rep..

[31] Shimokawa A., Tonooka Y., Matsumoto M., Ara H., Suzuki H., Yamauchi N., Shigyo M. (2014). Effect of alternating red and blue light irradiation generated by light emitting diodes on the growth of leaf lettuce. BioRxiv.

[32] Dougher T.A., Bugbee B. (2001). Differences in the response of wheat, soybean and lettuce to reduced blue radiation. Photochem. Photobiol..

[33] Wang J., Lu W., Tong Y., Yang Q. (2016). Leaf morphology, photosynthetic performance, chlorophyll fluorescence, stomatal development of lettuce (*Lactuca sativa* L.) exposed to different ratios of red light to blue light. Front. Plant Sci..

[34] Zha L., Liu W., Zhang Y., Zhou C., Shao M. (2019). Morphological and physiological stress responses of lettuce to different intensities of continuous light. Front. Plant Sci..

[35] Bailey-Serres J., Lee S.C., Brinton E. (2012). Waterproofing crops: Effective flooding survival strategies. Plant Physiol..

[36] van Dongen J.T., Licausi F. (2015). Oxygen sensing and signaling. Annu. Rev. Plant Biol..

[37] Considine M.J., Diaz-Vivancos P., Kerchev P., Signorelli S., Agudelo-Romero P., Gibbs D.J., Foyer C.H. (2017). Learning to breathe: Developmental phase transitions in oxygen status. Trends Plant Sci..

[38] Kim K., Kim C., Yoo J., Kim J.R., Kim Y.H., Lee S.E. (2023). Phosphine gas in the dark induces severe phytotoxicity in *Arabidopsis thaliana* by increasing a hypoxia stress response and disrupting the energy metabolism: Transcriptomic approaches. J. Hazard. Mater..

[39] Eysholdt-Derzsó E., Sauter M. (2019). Hypoxia and the group VII ethylene response transcription factor HRE2 promote adventitious root elongation in Arabidopsis. Plant Biol..

[40] Eysholdt-Derzsó E., Renziehausen T., Frings S., Frohn S., von Bongartz K., Igisch C.P., Mann J., Häger L., Macholl J., Leisse D. (2023). Endoplasmic reticulum–bound ANAC013 factor is cleaved by RHOMBOID-LIKE 2 during the initial response to hypoxia in *Arabidopsis thaliana*. Proc. Natl. Acad. Sci. USA.

[41] Riga P., Benedicto L., Gil-Izquierdo Á., Collado-González J., Ferreres F., Medina S. (2019). Diffuse light affects the contents of vitamin C, phenolic compounds and free amino acids in lettuce plants. Food Chem..

[42] Chen X.L., Li Y.L., Wang L.C., Yang Q.C., Guo W.Z. (2022). Responses of butter leaf lettuce to mixed red and blue light with extended light/dark cycle period. Sci. Rep..

[43] Chen X.L., Li Y.L., Wang L.C., Guo W.Z. (2021). Red and blue wavelengths affect the morphology, energy use efficiency and nutritional content of lettuce (*Lactuca sativa* L.). Sci. Rep..

[44] Ohashi-Kaneko K., Takase M., Kon N., Fujiwara K., Kurata K. (2007). Effect of light quality on growth and vegetable quality in leaf lettuce, spinach and komatsuna. Environ. Control Biol..

[45] Percudani R., Peracchi A. (2003). A genomic overview of pyridoxal-phosphate-dependent enzymes. EMBO Rep..

[46] Havaux M., Ksas B., Szewczyk A., Rumeau D., Franck F., Caffarri S., Triantaphylidès C. (2009). Vitamin B6 deficient plants display increased sensitivity to high light and photo-oxidative stress. BMC Plant Biol..

[47] Jain S.K., Lim G. (2001). Pyridoxine and pyridoxamine inhibits superoxide radicals and prevents lipid peroxidation, protein glycosylation, and (Na++ K+)-ATPase activity reduction in high glucose-treated human erythrocytes. Free Radic. Biol. Med..

[48] Stocker P., Lesgards J.F., Vidal N., Chalier F., Prost M. (2003). ESR study of a biological assay on whole blood: Antioxidant efficiency of various vitamins. Biochim. Biophys. Acta (BBA)-Gen. Subj..

[49] Kannan K., Jain S.K. (2004). Effect of vitamin B6 on oxygen radicals, mitochondrial membrane potential, and lipid peroxidation in H_2_O_2_-treated U937 monocytes. Free Radic. Biol. Med..

[50] Endo N., Nishiyama K., Okabe M., Matsumoto M., Kanouchi H., Oka T. (2007). Vitamin B6 suppresses apoptosis of NM-1 bovine endothelial cells induced by homocysteine and copper. Biochim. Biophys. Acta.

[51] Danon A., Miersch O., Felix G., op den Camp R.G., Apel K. (2005). Concurrent activation of cell death-regulating signaling pathways by singlet oxygen in *Arabidopsis thaliana*. Plant J..

[52] Edreva A., Velikova V., Tsonev T., Dagnon S., Gürel A., Aktaş L., Gesheva E. (2008). Stress-protective role of secondary metabolites: Diversity of functions and mechanisms. Gen. Appl. Plant Physiol..

[53] Rejeb I.B., Pastor V., Mauch-Mani B. (2014). Plant responses to simultaneous biotic and abiotic stress: Molecular mechanisms. Plants.

[54] Carvalho R.F., Takaki M., Azevedo R.A. (2011). Plant pigments: The many faces of light perception. Acta Physiol. Plant.

[55] Zoratti L., Karppinen K., Luengo Escobar A., Häggman H., Jaakola L. (2014). Light-controlled flavonoid biosynthesis in fruits. Front. Plant Sci..

[56] Zhou G.L., Zhu P. (2020). De novo transcriptome sequencing of *Rhododendron molle* and identification of genes involved in the biosynthesis of secondary metabolites. BMC Plant Biol..

[57] Nagano S., Mori N., Tomari Y., Mitsugi N., Deguchi A., Kashima M., Tezuka A., Nagano A.J., Usami H., Tanabata T. (2022). Effect of differences in light source environment on transcriptome of leaf lettuce (*Lactuca sativa* L.) to optimize cultivation conditions. PLoS ONE.

[58] Pertea M., Pertea G.M., Antonescu C.M., Chang T.C., Mendell J.T., Salzberg S.L. (2015). StringTie enables improved reconstruction of a transcriptome from RNA-seq reads. Nat. Biotechnol..

[59] Roberts A., Pimentel H., Trapnell C., Pachter L. (2011). Identification of novel transcripts in annotated genomes using RNA-Seq. Bioinformatics.

[60] Love M.I., Huber W., Anders S. (2014). Moderated estimation of fold change and dispersion for RNA-seq data with DESeq2. Genome Biol..

[61] Lohse M., Nagel A., Herter T., May P., Schroda M., Zrenner R., Tohge T., Fernie A.R., Stitt M., Usadel B. (2014). Mercator: A fast and simple web server for genome scale functional annotation of plant sequence data. Plant Cell Environ..

[62] Kanehisa M., Goto S. (2000). KEGG: Kyoto encyclopedia of genes and genomes. Nucleic Acids Res..

[63] Thimm O., Bläsing O., Gibon Y., Nagel A., Meyer S., Krüger P., Selbig J., Müller L.A., Rhee S.Y., Stitt M. (2004). MAPMAN: A user-driven tool to display genomics data sets onto diagrams of metabolic pathways and other biological processes. Plant J..

[64] State Administration for Market Regulation. https://www.samr.gov.cn.

[65] Chen X., Yang B., Huang W., Wang T., Li Y., Zhong Z., Yang L., Li S., Tian J. (2018). Comparative proteomic analysis reveals elevated capacity for photosynthesis in polyphenol oxidase expression-silenced *Clematis terniflora* DC. leaves. Int. J. Mol. Sci..

[66] Wang L., Lu W., Ran L., Dou L., Yao S., Hu J., Fan D., Li C., Luo K. (2019). R2R3-MYB transcription factor MYB 6 promotes anthocyanin and proanthocyanidin biosynthesis but inhibits secondary cell wall formation in *Populus tomentosa*. Plant J..

